# Effect of 9-month Pilates program on sagittal spinal curvatures and hamstring extensibility in adolescents: randomised controlled trial

**DOI:** 10.1038/s41598-020-66641-2

**Published:** 2020-06-19

**Authors:** Noelia González-Gálvez, Pablo Jorge Marcos-Pardo, Henry Trejo-Alfaro, Raquel Vaquero-Cristóbal

**Affiliations:** 1 Research Group on Health, Physical Activity, Fitness and Motor Behaviour (GISAFFCOM), Catholic University of San Antonio of Murcia (UCAM), Murcia, Spain; 2 Research Group on Prevention of Lesions in Sports, Catholic University of San Antonio of Murcia (UCAM), Murcia, Spain; 3 Present Address: Faculty of Sports, Catholic University of San Antonio of Murcia (UCAM), Av. de los Jerónimos, 135, 30107 Murcia, Spain

**Keywords:** Human behaviour, Quality of life, Therapeutics

## Abstract

The percentage of spine misalignment increases during the childhood and adolescence stages. The Pilates method has been associated with an improvement in the sagittal spine disposition, but no studies have been conducted on adolescents. Therefore, the present study aimed to evaluate the effectiveness of a 9-month Pilates exercise program (PEP) on hamstring extensibility and sagittal spinal curvatures on adolescents. This randomised controlled trial included 236 adolescents. The experimental group (EG) received a PEP (9 months, 2 sessions/week, 15 minutes/session). The control group (CG) did not receive any intervention. Hamstring extensibility was measured with the passive and active straight leg raise and toe-touch tests. Sagittal spinal curvatures and pelvic tilt was assessed in relaxed standing, active alignment and toe-touch test positions. The EG had significant changes in hamstring extensibility, lumbar curvature and pelvic tilt in standing sagittal curvature. The CG became significantly worse in thoracic kyphosis in standing. This study provides evidence of nine-months of a PEP increased the hamstring extensibility; averted the increase of the thoracic curvature, and decreased the curvature of the lumbar lordosis and pelvic tilt in standing position; avoided a greater increase of thoracic curvature in active alignment in standing position; and avoided the increase of thoracic curvature in trunk flexion.

## Introduction

Adolescence is a critical period for sagittal disposition, as several longitudinal studies have found that there is an increase in thoracic kyphosis especially in men; lumbar lordosis especially in women; and anterior pelvic tilt during this developmental stage^1,2^, although some cross-sectional studies have found that the changes during childhood and adolescence can be dependent on sex and anthropometric variables^3^. As a result, the percentage of misalignment increases during the childhood and adolescence stages^4^. Scientific evidence show that could be developing different injuries by spine misalignment in standing relaxing, trunk flexion and so on position. These injuries could be disc hernias, spondylolisthesis, disc hernias^5^, but also increases intradiscal pressure^6^, viscoelastic deformation^7,8^ and back pain^9,10^.

Several factors are associated with hyperkyphosis such as low values of hamstring extensibility and a lack of paravertebral strength in standing position^11,12^ and trunk flexion^13^ positions. In addition, it has been shown a lumbar kyphosis in maximal trunk flexion with knees extended positions^12^. Besides, coxofemoral joint has been associated with other injuries. Specifically, a lack of coxofemoral join mobility has been associated with the injuries previous mention and with patellar tendinopathy, patellofemoral pain or myotendinous junction lesions^14^.

Some research studies have shown improvements in hamstring extensibility after a specific extensibility program in adolescents^15–17^. However, although a systematic practice of some sports can induce changes in the spine disposition in this stage^18,19^, there are few studies that have investigated the effectiveness of a specific exercise program based on stretching and strengthening that can induce changes in spinal sagittal curvature in adolescents^20–22^. However, these few studies involved adolescents with non-structural sagittal spine curvatures that were more than normal and included a short intervention (8 weeks), so a decrease of the effects after a period of detraining is expected^23^, thus, maintaining the program during the academic year but with a lower volume of training is recommended^24^.

The Pilates method has been associated with an improvement in the sagittal spine disposition, flexibility and strength and a reduction in back pain and disability^25–27^. This method has been demonstrated to be effective on the two fitness factors associated with spine misalignment in adolescents: hamstring extensibility and trunk endurance^28,29^. Some studies have found that the practice of the Pilates method significantly reduces the thoracic and lumbar curvatures^27^, but no studies have been conducted on adolescents. Therefore, the objective of this study was to evaluate the effectiveness of a 9-month Pilates exercise program on hamstring extensibility and sagittal spinal curvatures in adolescents.

## Results

### Intragroup changes in hamstring extensibility and sagittal spinal curvature

There was significant increase in the values reached in the toe-touch (TT) test, the passive straight leg raise (P-SLR) (right and left), and the active straight leg raise (A-SLR) (right and left); a significant reduction was found in the lumbar lordosis and pelvic tilt both in standing position, pelvic tilt in active alignment in standing position; and a significant increase of the lumbar kyphosis in the TT test in the experimental group (EG), with effect size between low-moderate and very high. There was a significant increase of the thoracic kyphosis curvature in standing position and in the TT test in the control group (CG), with a moderate or high effect size. There was a significant increase of the thoracic kyphosis in active alignment of spinal curvatures in standing position in both groups, but the significance was high in CG, and the effect size was low for EG and moderate for CG (Table 1).

**Table 1:** Differences pre- to post- test (intra-groups) for sagittal spine curvatures and hamstring extensibility.

Test	Group	Pre-intervention (M ± SD)	Post-intervention (M ± SD)	Difference post-pre (M ± SD)	p-value	CI 95% (Mpost-Mpre)	ES
Toe-touch test	EG	−8.09 ± 8.81	−6.25 ± 9.99	2.04 ± 5.49	0.000	0.89;3.13	0.21
	CG	−11.66 ± 9.47	−11.30 ± 9.46	0.36 ± 6.56	0.602	−0.82;1.42	0.04
**Passive straight leg raise**							
Right	EG	82.79 ± 14.93	96.81 ± 19.05	13.95 ± 12.53	0.000	9.90;16.74	0.93
	CG	80.36 ± 15.49	84.02 ± 15.55	3.53 ± 10.63	0.001	−0.28;6.00	0.23
Left	EG	81.45 ± 14.57	94.58 ± 17.62	12.99 ± 13.25	0.000	9.90;16.74	0.90
	CG	80.33 ± 14.55	82.02 ± 16.00	1.73 ± 9.90	0.123	−0.28;6.00	0.12
**Active straight leg raise**							
Right	EG	69.73 ± 14.18	76.75 ± 17.71	6.98 ± 12.81	0.000	4.97;9.25	0.49
	CG	64.83 ± 13.90	65.79 ± 15.22	.88 ± 10.31	0.332	−1.08;3.21	0.07
Left	EG	68.58 ± 12.95	75.55 ± 17.43	6.85 ± 13.91	0.000	4.81;9.25	0.53
	CG	65.16 ± 13.63	63.03 ± 14.99	−2.31 ± 9.85	0.056	−4.40;0.05	0.16
**Relaxed standing position**							
Thoracic curve	EG	37.86 ± 10.44	38.39 ± 9.41	0.53 ± 10.80	0.544	−1.40;2.65	0.05
	CG	32.81 ± 11.54	38.80 ± 9.67	5.98 ± 11.40	0.000	4.03;8.10	0.52
Lumbar curve	EG	−36.03 ± 6.58	−32.40 ± 7.10	3.63 ± 6.86	0.000	1.96;4.87	0.55
	CG	−34.47 ± 6.82	−33.16 ± 7.12	1.31 ± 8.88	0.060	−0.05;2.86	0.19
Pelvic tilt	EG	25.97 ± 6.50	23.72 ± 6.77	−2.25 ± 7.14	0.008	−3.64;−0.54	0.34
	CG	24.58 ± 6.77	24.46 ± 7.25	−0.12 ± 9.87	0.611	−1.96;1.15	0.02
**Active alignment in standing position**							
Thoracic curve	EG	24.83 ± 12.38	27.50 ± 11.43	2.52 ± 13.36	0.044	0.06;5.11	0.21
	CG	20.23 ± 13.39	26.88 ± 10.78	6.65 ± 13.78	0.000	4.16;9.22	0.49
Lumbar curve	EG	−32.70 ± 9.98	−31.73 ± 7.44	0.897 ± 10.61	0.064	−1.19;2.81	0.10
	CG	−30.66 ± 10.47	−30.16 ± 8.87	0.50 ± 12.08	0.237	−1.81;2.21	0.05
Pelvic tilt	EG	26.07 ± 7.22	23.85 ± 8.82	−2.26 ± 9.73	0.021	−4.00;−.32	0.31
	CG	24.67 ± 8.92	24.23 ± 7.18	−0.44 ± 10.23	0.455	−2.54;1.14	0.05
**Toe-touch test position**							
Thoracic curve	EG	48.72 ± 11.71	51.10 ± 11.98	2.38 ± 13.43	0.063	−0.11;4.37	0.20
	CG	51.42 ± 11.47	55.47 ± 11.07	4.11 ± 10.66	0.000	1.80;6.31	0.35
Lumbar curve	EG	31.25 ± 7.59	32.80 ± 9.21	1.55 ± 9.04	0.049	0.00;3.16	0.20
	CG	31.50 ± 8.57	30.84 ± 8.26	−0.52 ± 7.92	0.579	−2.03;1.13	0.08
Pelvic tilt	EG	66.72 ± 14.38	67.53 ± 17.48	0.81 ± 12.86	0.502	−1.41;2.87	0.06
	CG	61.69 ± 14.80	63.84 ± 16.12	1.96 ± 10.16	0.087	−0.27;4.02	0.14

EG: Experimental Group; CG: Control group; M: Mean; SD: Standard Deviation; ES: Effect Size.

### Differences between groups in the change

There were significant differences between groups for the pre- to post- test changes in the P-SLR, A-SLR and TT tests, thoracic kyphosis in both relaxed standing position and active alignment of spinal curvatures in standing position (Table 2).

**Table 2:** Differences between groups in the pre- post- test change for sagittal spine curvature and hamstring extensibility.

Test	Group	Difference post-pre (Mean ± SD)	Diff. Post-pre EG - Diff. Post-pre GC	CI 95% (Diff. Post-pre EG - Diff. Post-pre GC)	F/Z	p-value	ES
Toe-touch test	EG	2.04 ± 5.49	1.67	0.122; 3.23	4.551	0.034	0.020
	CG	0.36 ± 6.56					
**Passive straight leg raise**							
Right	EG	13.95 ± 12.53	10.41	7.41;13.41	39.469	0.000	0.148
	CG	3.53 ± 10.63					
Left	EG	12.99 ± 13.25	11.26	8.25;14.27	51.591	0.000	0.185
	CG	1.73 ± 9.90					
**Active straight leg raise**							
Right	EG	6.98 ± 12.81	6.10	3.10; 9.10	15.458	0.000	0.064
	CG	0.88 ± 10.31					
Left	EG	6.85 ± 13.91	9.15	6.04;12.25	33.26	0.000	0.128
	CG	−2.31 ± 9.85					
**Relaxed standing position**							
Thoracic curve	EG	0.53 ± 10.80	−5.45	−8.30;−2.60	13.906	0.000	0.058
	CG	5.98 ± 11.40					
Lumbar curve	EG	3.63 ± 6.86	2.32	0.28;4.35	3.707	0.055	0.016
	CG	1.31 ± 8.88					
Pelvic tilt	EG	−2.25 ± 7.14	−2.12	−4.33;0.08	2.297	0.131	0.010
	CG	−0.12 ± 9.87					
**Active alignment in standing position**							
Thoracic curve	EG	2.52 ± 13.36	−4.13	−7.62;−0.64	5.111	0.025	0.022
	CG	6.65 ± 13.78					
Lumbar curve	EG	0.95 ± 10.61	0.44	−2.47;3.37	0.564	0.573	0.04
	CG	0.50 ± 12.08					
Pelvic tilt	EG	−2.26 ± 9.73	−1.81	−4.38;0.75	1.225	0.270	0.005
	CG	−0.44 ± 10.23					
**Toe-touch test position**							
Thoracic curve	EG	2.38 ± 13.43	−1.72	−4.84;1.38	1.433	0.233	0.006
	CG	4.11 ± 10.66					
Lumbar curve	EG	1.55 ± 9.04	2.07	−0.11;4.25	3.199	0.075	0.014
	CG	−0.52 ± 7.92					
Pelvic tilt	EG	0.81 ± 12.86	−1.14	−4.12;1.83	0.555	0.457	0.002
	CG	1.96 ± 10.16					

EG: Experimental Group; CG: Control group; M: Mean; SD: Standard Deviation; ES: Effect Size.

## Discussion

### Effect of the Pilates exercise program on hamstring extensibility

Significant improvements in hamstring extensibility were observed from baseline to 9 months in the EG after the Pilates program in all of the tests used. Also, a significant difference in the change between groups was reported for all the measurements. In accordance with the present results, previous studies with adolescents in physical education classes have demonstrated that a Pilates exercise program lasting 60 minutes/session, 2 sessions per week for 6 weeks improved hamstring extensibility^28,29^.

A greater number of studies have implemented a specific extensibility program into physical education classes and have assessed their effect on hamstring extensibility in adolescents. These studies used 3–7 minutes/session, two weekly sessions, and lasted between 5 and 8 weeks, showing significant improvements after the experimental period^15–17^. These results are connected with the findings of the present study, which has the advantage that the Pilates method is a more complete technique that not only improves extensibility but also increases muscular endurance^28,30^, and these are two fitness factors that are mainly associated with spine misalignment^9,11–13^.

### Effect of the Pilates exercise program on sagittal spinal curvature

There are no previous studies that have implemented a Pilates exercise program and assessed its effect on the sagittal spinal curvature of adolescents. The results of this study indicate that adolescents who took part in a Pilates method program did not show changes in thoracic curvature and had a significant reduction of the lumbar curvature and pelvic tilt in relaxed standing position. The CG had a significant increase in their thoracic curve in this position. The difference in the change between groups was significant for the thoracic curve.

This finding broadly supports the work from other studies in this area, linking the practice of the Pilates method, 60–75 minutes/session, 2 or 3 times per week for 10 to 30 weeks, with a decrease of the thoracic^27,31^ and lumbar curves^27^ in adults and older adults. In the present study, significant differences were found only for the lumbar curvature. A possible explanation for this could be that the thoracic curve increases with age, especially during the growing stage^32^.

This result could also be explained by the fact that the Pilates method has been associated with an improvement in the stability of the spine, hamstring extensibility and trunk strength in adolescents^28,29^, factors associated with spine misalignment^9,11–13^.

In fact, studies that performed a systematic exercise program based on stretching and strengthening exercises, 2–3 days per week, 15–60 minutes per session, for 8 weeks, with male^21^ and female adolescents^20,22^ with hyperkyphosis^21,22^ and hyperlordosis^20,21^ have found a significant decrease in thoracic and lumbar curvatures, with our results in line with these studies. The Pilates method program reduced lumbar curvature and averted an increase of the thoracic curve. This suggests that the Pilates method could be an effective exercise for maintaining the curve within the normal range or for reducing it.

Also, the reduction of the pelvic tilt after a 9-month Pilates exercise programme is completely justified. Other researches have connected the lumbar curvature with the pelvic tilt, showing high curves with high pelvic tilts and vice versa^33,34^. This correlation was found in the present study as well.

Both groups reported a significant increase in the thoracic kyphosis curve in active alignment in standing position, which is frequent in the developmental stage associated with changes in the sagittal spine disposition of the vertebrae^1,2,4^, although a greater change was found in the CG than in the EG. According to these results, two 15-minute sessions per week of Pilates program cannot completely stop the changes in the sagittal spine disposition of the vertebrae, but it can slow it down. Further work is required to establish the relation between training volume and its effects in active alignment in standing position. Also, the EG showed a significant reduction in pelvic tilt in this position. The relevance of this resides in the connection of the active alignment in standing position and the severity of the misalignment or its possibility of its severity increasing. The misalignment of the spine that the participant could reduce voluntarily is considered as functional or attitudinal, although it is not always associated with a severe alteration. However, if the participant could not modify the curvature voluntarily, this is denominated as a structural misalignment and usually shows wedges and alterations in the bone structures^35^. Therefore, maintaining the mobility of the spinal sagittal curvatures and showing a greater active alignment in the standing position will prove to be an improvement in the health of the spine^36^. During the growing period, especially at the peak height velocity period, the capacity to self-reduce the sagittal spine curves decreases^32^. Thus, it can be suggested that the practice of the Pilates method slows the decline in this ability that occurs with age.

The results of this study show that thoracic kyphosis in CG and lumbar kyphosis in EG significantly increase in the TT position. Muyor *et al*.^33^ also showed a significant decrease in the thoracic curve in the TT test position with a stretching program. People with hamstring shortening can let the pelvic retroversion be near a neutral position in maximal trunk position, which allows the lumbar curve to be increased. As a consequence, a full thoracic flexion is not needed^33^. Therefore, the present study confirms that a change in hamstring extensibility can be associated with sagittal disposition in maximal trunk positions.

Most studies have found improvements in the sagittal arrangement of the spine combined with a stretching and strengthening exercise in their programs. Therefore, and taking into account that both fitness factors are mainly associated with spine misalignment^5,12,13^, research is needed to learn about how these fitness factors separately or differently (with different loads) affect a Pilates method program on sagittal spinal curvatures in different positions.

It could be interesting for future studies to compare the effect of different frequencies and durations of Pilates method programs in adolescents in order to define the training parameters to consider depending on the objective: to prevent or to reduce a misalignment of the spine.

### Strength and limits

The viable exercise program is the first strength of this study. The exercise program presented in this research study only lasted 15 minutes, while most of studies cited used all the useful session time. Although these studies showed important improvements, sometimes these kinds of programs were difficult to implement because the Physical Education class has to deal with several other topics aside from Pilates. In addition, some of these studies implemented exercise programs for 6 weeks or less, and it has been confirmed that the fitness qualities gained after a specific program is lost in five^16^ or eight^37^ weeks in adolescents. Therefore, finding an exercise programme that could be implemented throughout the academic year is highly important in order to avoid the effect of detraining. Along this line, the protocol created in this study only uses a small part of the class session and this was enough to result in improvements; therefore, the exercise program used in the present study is viable and susceptible for its implementation throughout the entire academic year to improve hamstring extensibility and spinal sagittal curvatures and to avoid the negative effects of detraining.

Strong research methodologies were used in this study, such as a randomized clinical trial with blinded examiner. In addition, a large sample size was recorded to minimize the risk of bias, the exercise program implemented had a long duration, and included an evaluation of thoracic and lumbar curves in different positions.

In line with what has been presented, and due to the few previous studies conducted, with adolescents and a small sample, it should be noted that the results of this study are not conclusive. The study findings shed some light on how a Pilates method implemented during an academic year could improve hamstring extensibility and avoid the increase or reduce the spinal sagittal curvature of adolescents.

## Conclusion

Nine-months of a Pilates exercise program increased hamstring extensibility; averted the increase of the thoracic curvature, and decreased the curvature of the lumbar lordosis and pelvic tilt in standing position; averted a greater increase of the thoracic curvature in active alignment in standing position; and averted the increase of thoracic curvature in trunk flexion. This effectiveness suggests that a long-term Pilates method could be a viable and appropriate program that could be implemented in an academic year to improve hamstring extensibility and to maintain the sagittal curvatures within normal values of adolescents.

## Methods

The present study is a 9-month randomised controlled trial^38^. The trial design was registered with ClinicalTrial.gov (identifier: NCT03831867) and followed the Consolidated Standards of Reporting Trials (CONSORT) guidelines. All parents signed an informed consent form approved by the “Scientific and Ethical Committee” of the institution (code: TC4/17). All the experimental protocols were approved by the Ethics Committee of the Catholic University of San Antonio of Murcia (Spain) following the guidelines of the Helsinki Declaration.

This study was conducted at a school and sport science laboratory. Student were recruited from a high school in the Region of Murcia (Spain).

A total of 236 students participated (male = 124; female = 112) aged between 12 and 17 years old (average age: 13.15 ± 1.24 years old) and they were randomised into the EG (n = 118) and GC (n = 118).

The inclusion criteria were: (a) being in Compulsory Secondary Education; (b) physically active in school physical education sessions. The exclusion criteria were: a) missing more than one session of the program; b) presenting any neurological (muscular dystrophy, spina bifida, injuries to the spinal cord and brain, seizure disorders such as epilepsy, cancer such as brain tumours, infections such as meningitis), cardiovascular (hypertension, cerebrovascular disease, peripheral vascular disease, heart failure, rheumatic heart disease, congenital heart disease, cardiomyopathies), musculoskeletal (arthritis, gout, ankylosing spondylitis associated fragility fractures, traumatic fractures, inflammatory diseases, injuries, structural spinal pathology, orthopaedic pathology in hip, knee and/or feet) or metabolic alterations (dyslipidaemia, obesity, diabetes, high blood pressure) at the time of the measurement; (c) and not having practiced at least 60 minutes of moderate- to vigorous-intensity physical activity daily^39^ measured using the short version of the Physical Activity Questionnaire for Adolescents (PAQ-A)^40^, which had been translated and validated for Spanish adolescents^41^. The CONSORT 2010 flow diagram is shown in Supplementary Fig. 1.

The EG took part in a Pilates exercise program lasting 9 months. They performed two 15-minute sessions per week. The CG did not take part in any specific or structured exercise program, although they attended their regular physical education sessions.

The Pilates program was taught by the physical education teacher with a Pilates training certificate; and took place at school.

**Figure 1:**
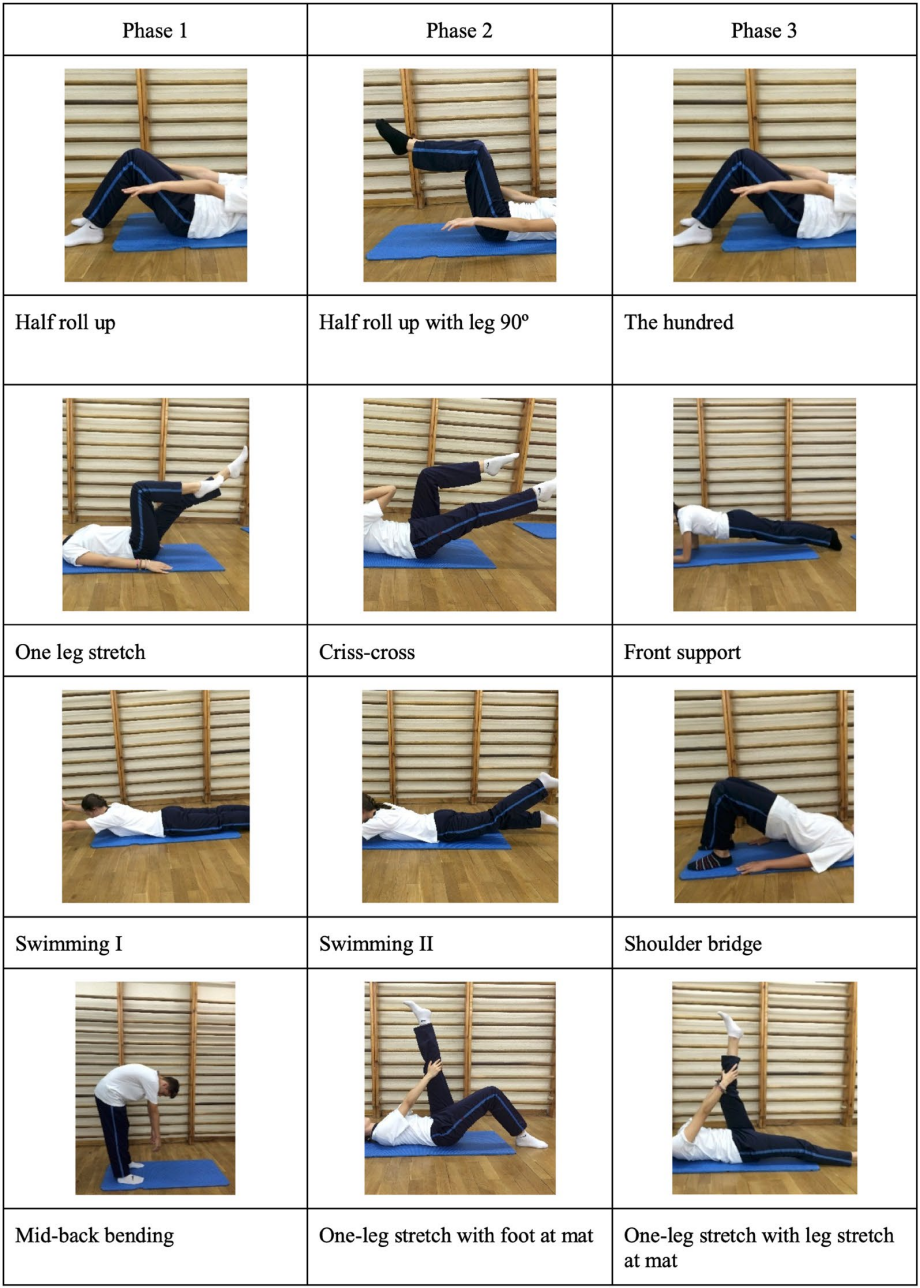
Pilates method exercise. *Four exercises are performed in phase 1: half roll-up (4 min, 3 sets, 12 reps, aim: strengthening the abdominals and torso stability), one-leg stretch (4 min, 2 sets, 12 reps, aim: strengthening the abdominals, torso stability, mobilization of the hip, and hamstring flexibility), swimming I (4 min, 2 sets, 12 reps, aim: back muscle strengthening and breathing cycle), and mid-back bending (3 min, 5 reps, aim: stretching back and hamstring muscle and relaxing); in phase 2: half roll-up with leg 90° (4 min, 3 set, 12 reps, aims: strengthening the abdominals and torso stability), criss-cross (4 min, 3 sets, 12 reps, aim: strengthening the abdominals, torso stability, neck strengthening; mobilization of the hip, and hamstring flexibility), swimming II (4 min, 2 sets, 12 reps, aim: back muscle strengthening and breathing cycle), and one-leg stretch with foot at mat (3 min, 2 sets, 30 sec/sets each leg, aims: stretching hamstring muscle and relaxing); phase 3: the hundred (4 min, 2 sets, 50 reps, aim: strengthening the abdominals, torso stability, and breathing cycle); front support (4 min, 2 sets, 20 secs, aim: strengthening the abdominals and back muscle, torso stability, and breathing cycle), shoulder bridge (4 min, 2 sets, 12 reps, aims: back muscle strengthening, spine mobilization, and hamstring flexibility), and one-leg stretch with leg stretch on mat (3 min, 3 sets, 30 secs with each leg, aim: stretching hamstring muscle and relaxing).

The Pilates method exercise protocol is described in Supplementary Table 1. It was divided into three phases. Each subsequent phase increased the exercise difficulty and incorporated more principles of the Pilates method. The exercises of the old phases were exchanged for the exercises of the new phase one by one in each session. Figure 1 shows the representation of the exercises.

All the measurements were performed by the same researchers in a single session between the hours of 10:00 and 14:00. No warm-up or stretching exercises were performed by the participants before the test measurements. The participants were examined barefoot. The laboratory temperature was standardized at 24 °C. There was a 5-minute rest between measurements.

In order to assess hamstring extensibility, P-SLR, A-SLR and TT tests were used.

For P-SLR and A-SLR, the participant was placed in the supine position on a stretcher with legs extended. The examiner placed a UNI-LEVEL inclinometer (Isomed, Inc., Portland, OR, USA) over the distal tibia, set to 0° to measure the inclination. The opposite pelvis and leg were in a straight position and an auxiliary tester stopped posterior tilt during the test^42^. For the P-SLR test, the examiner performed a slow and progressive coxofemoral joint bend of the examined leg, in order to reach the maximum hip flexion without any other movement, to avoid a compensation movement such as external rotation, abduction, etc. The movement was stopped when the participant manifested discomfort or pain in the popliteal gap or a pelvic retroversion was detected by the main researcher or the assistant, at which time the inclinometer was read. For the A-SLR test, the participant performed a slow and progressive, voluntary and active coxofemoral joint bend without help of the examiner, as much as the participant could. It was measured on both legs.

For the TT test, the participants stood on the measuring drawer, with knees extended and feet together. The participant performed the maximum bend of the trunk without bending the knees, with the arms and palms extended over the drawer ruler. The zero of the ruler coincided with the top surface of the drawer. Values that were above the surface of the drawer were considered negative, and those that remained below were positive. The measurement was recorded in centimetres^43^.

To assess the sagittal spinal curvatures and pelvic tilt, the SPINAL MOUSE SYSTEM (Idiag, Fehraltdorf, Switzerland) was used. The SPINAL MOUSE is an electronic device connected via Bluetooth to a computer. This system has been used to measure the sagittal spinal range, the angle of the thoracic curve, lumbar curve and pelvic tilt in different positions, in a non-invasive way. It is the best known and used instrument, with a high validity, intra-rater reliability (Intraclass Correlation Coefficients -ICCs-: 0.61–0.96) and inter-rater reliability (ICCs: 0.70–0.93)^44^. The sagittal spinal curvature was assessed in relaxed standing, active alignment of spinal curvatures in standing and TT test positions.

A SECA 878 scale (SECA, Germany) was used to measure body mass and a SECA 274 stadiometer (SECA, Germany) to measure height.

In connection with the standard deviation established for thoracic sagittal curvature of the spine measure with the SPINAL MOUSE in previous studies^33^, an estimated error of 2 degrees and a significance level of α = 0.01 were utilized, with a valid sample size for a confidence interval of 99% being 142.38. A total of 236 students completed the trial. The final sample size for each group in our study (EG = 118, CG = 118) provided a power of 99% and an estimated error of 1.55 degrees. The Rstudio 3.15.0 software was used to establish the sample size.

Following the initial evaluation, the participants were distributed into the EG and CG. A simple randomisation method (Microsoft Excel 2016) was used to allocate participants to the EG or CG. Group assignment was blinded to the examiner and staff who performed the statistical analysis.

After analysing the normality of variables (Kolmogorov-Smirnov test), a two-way ANOVA with repeated measures in 1 factor (time) was used to analyse inter- and intra-group differences and to analyse the interaction between groups and time. The Bonferroni post-hoc test was used to evaluate the statistical significance of the parametric variables. For non-parametric variables, the Wilcoxon signed rank was used to check intragroup changes and the Mann-Whitney test was used to check intergroup differences. Size effect was calculated, defined as low: r = 0.10; moderate: r = 0.30, high: r = 0.50; very high: r = 0.70^45^. Spearman’s rank correlation coefficient (rho) was used to assess the relationship between spinal sagittal curvatures in relaxed standing position and TT-test position. An error of p ≤ 0.05 was established. The statistical analysis was performed using the statistical package SPSS 21.0 for Windows.

## Supplementary information

Supplementary Figure S1.

Supplementary Table S1.
