# Supplementary material for: Effect of 9-month Pilates program on sagittal spinal curvatures and hamstring extensibility in adolescents: randomised controlled trial
Source: Sci Rep. 2020 Jun 19;10:9977. doi: 10.1038/s41598-020-66641-2 (PMC7305121; doi:10.1038/s41598-020-66641-2)
Supplement: Supplementary file 1 — Supplementary Figure S1. [file 41598_2020_66641_MOESM1_ESM.doc]

Effect of 9-month Pilates program on sagittal spinal curvatures and hamstring extensibility in adolescents: randomised controlled trial

Noelia González-Gálvez^1^; Pablo Jorge Marcos-Pardo^1*^; Henry Trejo-Alfaro^1^ and Raquel Vaquero-Cristóbal^2^.

^1^Grupo de Investigación en Salud, Actividad Física, Fitness y Comportamiento Motor (GISAFFCOM). Faculty of Sport, Catholic University of San Antonio (UCAM), Murcia, Spain.

^2^ Faculty of Sport, Catholic University of San Antonio (UCAM), Murcia, Spain.

Current Address: Faculty of Sport, Catholic University San Antonio of Murcia (UCAM), Av. de los Jerónimos, 135, 30107, Murcia (Spain).

*Correspondence to [pmarcos@ucam.edu](mailto:pmarcos@ucam.edu)

**Supplementary figure 1:** Consort flow diagram

**Allocation**

**Analysis**

**Follow-Up**

**Enrollment**

Assessed for eligibility (n=292)

Excluded (n=45)

♦  Not meeting inclusion criteria (n=29)

♦  Declined to participate (n=16)

Analysed (n=118)
♦ Excluded from analysis (give reasons) (n=0)

Lost to follow-up (changed school or reassigned from project) (n=2). Discontinued intervention (n=3).

Allocated to intervention (n=123)

♦ Received allocated intervention (n=123)

♦ Did not receive allocated intervention (give reasons) (n=0)

Lost to follow-up (changed school or reassigned from project) (n=1). Discontinued intervention (n=4). Leg injury (n=1)

Allocated to intervention (n=124)

♦ Received allocated intervention (n=124)

♦ Did not receive allocated intervention (give reasons) (n=0)

Analysed (n=118)
♦ Excluded from analysis (give reasons) (n=0)

Randomized (n=247)
