# Supplementary material for: Effect of 9-month Pilates program on sagittal spinal curvatures and hamstring extensibility in adolescents: randomised controlled trial
Source: Sci Rep. 2020 Jun 19;10:9977. doi: 10.1038/s41598-020-66641-2 (PMC7305121; doi:10.1038/s41598-020-66641-2)
Supplement: Supplementary file 2 — Supplementary Table S1. [file 41598_2020_66641_MOESM2_ESM.docx]

Effect of 9-month Pilates program on sagittal spinal curvatures and hamstring extensibility in adolescents: randomised controlled trial

Noelia González-Gálvez^1^; Pablo Jorge Marcos-Pardo^1*^; Henry Trejo-Alfaro^1^ and Raquel Vaquero-Cristóbal^2^.

^1^ Grupo de Investigación en Salud, Actividad Física, Fitness y Comportamiento Motor (GISAFFCOM). Faculty of Sport, Catholic University of San Antonio (UCAM), Murcia, Spain.

^2^ Faculty of Sport, Catholic University of San Antonio (UCAM), Murcia, Spain.

Current Address: Faculty of Sport, Catholic University San Antonio of Murcia (UCAM), Av. de los Jerónimos, 135, 30107, Murcia (Spain).

*Correspondence to [pmarcos@ucam.edu](mailto:pmarcos@ucam.edu)

**Supplementary table 1:** Pilates method exercises

|  | | | |
| --- | --- | --- | --- |
| Exercise | Time, min | Repetitions | Objectives and hints |
| Phase 1 - October, November and December | | | |
| Half roll-up | 4 | 3 sets × 12 reps | Strengthening the abdominals and torso stability |
| One-leg stretch | 4 | 2 sets × 12 reps | Strengthening the abdominals, torso stability, mobilization of the hip, and hamstring flexibility |
| Swimming I | 4 | 2 sets × 13 reps | Back muscle strengthening and breathing cycle |
| Mid-back bending | 3 | 5 reps | Stretching back and hamstring muscle and relaxing |
| Phase 2 (January, February and first half of March) | | | |
| Half roll-up with leg 90º | 4 | 3 sets × 12 reps | Strengthening the abdominals and torso stability |
| Criss-cross | 4 | 2 sets × 12 reps | Strengthening the abdominals, torso stability, neck strengthening, mobilization of the hip, and hamstring flexibility |
| Swimming II | 4 | 2 sets × 12 breathing cycle | Back muscle strengthening and breathing cycle |
| One-leg stretch with foot at mat | 3 | 2 sets × 30 sec (each leg) | Stretching hamstring muscle and relaxing |
| Phase 3 (second half of March, April and May) | | | |
| The hundred | 4 | 2 sets × 50 reps | Strengthening the abdominals, torso stability, and breathing cycle |
| Front support | 4 | 2 sets × 20 sec | Strengthening the abdominals and back muscle, torso stability, and breathing cycle |
| Shoulder bridge | 4 | 2 sets × 12 reps | Back muscle strengthening, spine mobilization, hamstring flexibility |
| One-leg stretch with leg stretch at mat | 3 | 3 sets × 30 sec (each leg) | Stretching hamstring muscle and relaxing |
| min = minutes; reps = repetitions; sec = seconds. | | | |
